# Development assistance for community health workers in 114 low- and middle-income countries, 2007–2017

**DOI:** 10.2471/BLT.19.235499

**Published:** 2019-11-01

**Authors:** Chunling Lu, Daniel Palazuelos, Yiqun Luan, Sonia Ehrlich Sachs, Carole Diane Mitnick, Joseph Rhatigan, Henry B Perry

**Affiliations:** a Brigham & Women's Hospital, Harvard Medical School, 641 Huntington Ave, Boston, Massachusetts 02115, United States of America (USA).; b The Heller School for Social Policy and Management, Brandeis University, Waltham, USA.; c Earth Institute, Columbia University, New York, USA.; d Department of Global Health and Social Medicine, Harvard Medical School, Boston, USA.; e Department of International Health, Johns Hopkins University, Baltimore, USA.

## Abstract

**Objective:**

To estimate the level and trend of development assistance for community health worker-related projects in low- and middle-income countries between 2007 and 2017.

**Methods:**

We extracted data from the Organisation for Economic Co-operation and Development’s creditor reporting system on aid funding for projects to support community health workers (CHWs) in 114 countries over 2007–2017. We produced estimates for projects specifically described by relevant keywords and for projects which could include components on CHWs. We analysed the pattern of development assistance by purpose, donors, recipient regions and countries, and trends over time.

**Findings:**

Between 2007 and 2017, total development assistance targeting CHW projects was around United States dollars (US$) 5 298.02 million, accounting for 2.5% of the US$ 209 277.99 million total development assistance for health. The top three donors (Global Fund to Fight AIDS, Tuberculosis and Malaria, the government of Canada and the government of the United States of America) provided a total of US$ 4 350.08 million (82.1%) of development assistance for these projects. Sub-Saharan Africa received a total US$ 3 717.93 million, the largest per capita assistance over 11 years (US$ 0.39; total population: 9 426.25 million). Development assistance to projects that focused on infectious diseases and child and maternal health received most funds during the study period.

**Conclusion:**

The share of development assistance invested in the CHW projects was small, unstable and decreasing in recent years. More research is needed on tracking government investments in CHW-related projects and assessing the impact of investments on programme effectiveness.

## Résumé

**Objectif:**

Estimer le montant et l'évolution de l'aide au développement portant sur des projets relatifs aux agents de santé communautaires dans les pays à revenu faible et intermédiaire, entre 2007 et 2017.

**Méthodes:**

Nous avons extrait des données du Système de notification des pays créanciers de l'Organisation de coopération et de développement économiques concernant le financement de l'aide apportée à des projets axés sur les agents de santé communautaires dans 114 pays, de 2007 à 2017. Nous avons obtenu des estimations pour les projets spécifiquement décrits par certains mots clés et pour les projets dont certaines parties pouvaient concerner les agents de santé communautaires. Nous avons analysé les tendances de l'aide au développement par objectif, donateurs, régions et pays destinataires, ainsi que son évolution dans le temps.

**Résultats:**

Entre 2007 et 2017, le total de l'aide au développement versée à des projets portant sur les agents de santé communautaires s'est élevé à environ 5298,02 millions de dollars des États-Unis ($ US), soit 2,5% des 209 277,99 millions de $ US d'aide au développement totale allouée à la santé. Les trois principaux donateurs (Fonds mondial de lutte contre le sida, la tuberculose et le paludisme, gouvernement du Canada et gouvernement des États-Unis d'Amérique) ont versé un total de 4350,08 millions de $ US (82,1%) d'aide au développement pour ces projets. L'Afrique subsaharienne a reçu un total de 3717,93 millions de $ US, soit l'aide par habitant la plus importante sur les 11 années (0,39 $ US; population totale: 9426,25 millions). Durant la période examinée, la majorité des fonds alloués à l'aide au développement étaient destinés à des projets portant sur les maladies infectieuses et la santé maternelle et infantile.

**Conclusion:**

La part de l'aide au développement investie dans des projets axés sur les agents de santé communautaires était faible, irrégulière et en baisse ces dernières années. De plus amples recherches sont nécessaires pour suivre les investissements du gouvernement dans des projets concernant les agents de santé communautaires et évaluer l'impact de ces investissements sur l'efficacité des programmes.****

## Resumen

**Objetivo:**

Estimar el nivel y la tendencia de la asistencia para el desarrollo destinada a proyectos relacionados con los trabajadores de salud de la comunidad en países de bajos y medianos ingresos entre 2007 y 2017.

**Métodos:**

Se extrajeron datos del sistema de información de acreedores de la Organización para la Cooperación y el Desarrollo Económicos sobre la financiación de la ayuda para proyectos de apoyo a los trabajadores de salud de la comunidad (community health workers, CHW) en 114 países entre 2007 y 2017. Se elaboraron estimaciones para los proyectos descritos específicamente mediante palabras clave pertinentes y para los proyectos que podrían incluir componentes sobre los CHW. Se analizó la pauta de la asistencia para el desarrollo por finalidad, donantes, regiones y países receptores, y las tendencias a lo largo del tiempo.

**Resultados:**

Entre 2007 y 2017, el total de la asistencia para el desarrollo destinada a proyectos de CHW fue de unos 5 298,02 millones de dólares estadounidenses (USD), lo que representa el 2,5 % del total de la asistencia para el desarrollo destinada a la salud, que ascendió a 209 277,99 millones de USD. Los tres principales donantes (el Fondo Mundial de Lucha contra el SIDA, la Tuberculosis y la Malaria, el Gobierno de Canadá y el Gobierno de los Estados Unidos de América) aportaron un total de 4 350,08 millones de USD (82,1 %) de la asistencia para el desarrollo destinada a estos proyectos. El África subsahariana recibió un total de 3 717,93 millones de USD, la mayor asistencia per cápita en 11 años (0,39 USD; población total: 9 426,25 millones). La asistencia para el desarrollo de proyectos centrados en las enfermedades infecciosas y la salud materno infantil recibió la mayor parte de los fondos durante el periodo de estudio.

**Conclusión:**

La contribución de la asistencia para el desarrollo invertida en los proyectos de los CHW fue pequeña, inestable y decreciente en los últimos años. Se necesita más investigación sobre el seguimiento de las inversiones gubernamentales en proyectos relacionados con los CHW y la evaluación del impacto de las inversiones en la eficacia de los programas.

## ملخص

**الغرض:**

تقدير مستوى واتجاه المساعدة الإنمائية لمشروعات العاملين بالقطاع الصحي المجتمعي في البلدان ذات الدخل المنخفض والمتوسط، بين عامي 2007 و2017.

**الطريقة:**

قمنا باستخراج البيانات من نظام إعداد التقارير للمشاركين في منظمة التعاون الاقتصادي والإنمائي بشأن تمويل المساعدات لمشروعات دعم العاملين بالقطاع الصحي المجتمعي (CHW) في 114 دولة خلال الفترة من 2007 إلى 2017. وأصدرنا تقديرات للمشروعات الموصوفة بشكل محدد عن طريق كلمات مفتاحية مناسبة، وللمشروعات التي يمكن أن تشمل مكونات من العاملين بالقطاع الصحي المجتمعي. قمنا بتحليل نمط المساعدة الإنمائية حسب الغرض، والجهات المانحة، والمناطق والبلدان المستفيدة، والاتجاهات على مر الزمن.

**النتائج:**

بين عامي 2007 و2017، بلغ إجمالي المساعدة الإنمائية التي تستهدف مشروعات العاملين بالقطاع الصحي المجتمعي، حوالي 5298.02 مليون دولاراً أمريكياً (دولار الولايات المتحدة)، وهو ما يمثل 25% من 209277.99 مليون دولاراً أمريكياً هو إجمالي المساعدة الإنمائية للصحة. قدمت أكبر ثلاث جهات مانحة (الصندوق العالمي لمكافحة الإيدز والسل والملاريا، وحكومة كندا، وحكومة الولايات المتحدة الأمريكية)، ما يصل مجموعه إلى 4350.08 مليون دولاراً أمريكياً (82.1%) من المساعدة الإنمائية لهذه المشروعات. حصلت جنوب الصحراء الأفريقية على إجمالي 3717.93 مليون دولارًا أمريكياً، وهي أكبر مساعدة للفرد على مدى 11 عاماً (0.39 دولاراً أمريكياً؛ إجمالي السكان: 9426.25 مليون). إن المساعدة الإنمائية للمشروعات التي ركزت على الأمراض المعدية وصحة الطفل والأم، حصلت على معظم الأموال خلال فترة الدراسة.

**الاستنتاج:**

كانت حصة المساعدة الإنمائية التي تم استثمارها في مشروعات العاملين بالقطاع الصحي المجتمعي، صغيرة وغير مستقرة ومتناقصة في السنوات الأخيرة. هناك حاجة لإجراء مزيد من البحث على تتبع الاستثمارات الحكومية في المشروعات المتعلقة بالعاملين بالقطاع الصحي المجتمعي، وتقييم تأثير الاستثمارات على فعالية البرنامج. ****

## 摘要

**目的:**

旨在评估与 2007-2017 年中低收入国家社区卫生工作者相关项目的发展援助水平及趋势。

**方法:**

我们从经济合作与发展组织的债权人报告系统中提取 2007-2017 年间 114 个国家支持社区卫生工作者 (CHW) 项目的援助资金数据。我们对相关关键词具体描述的项目以及可能由社区卫生工作者组成的项目进行了评估。我们按目的、捐助者、受援区域和国家以及长期趋势分析了发展援助的模式。

**结果:**

2007 年至 2017 年期间,针对社区卫生工作者项目的发展援助总额约为 52.9802 亿美元,占卫生发展援助总额 2092.7799 亿美元的 2.5%。前三大捐助者(全球抗击艾滋病、结核和疟疾基金、加拿大政府和美利坚合众国政府)为这些项目提供了总计 43.5008 亿美元 (82.1%) 的发展援助资金。撒哈拉以南非洲地区共获得 37.1793 亿美元的援助,是 11 年来人均受援助最多的地区(平均每人 0.39 美元;总额:94.2625 亿美元)。在研究期间,大部分资金被分配到了以传染病和母婴健康为重点的项目的发展援助。

**结论:**

投资于社区卫生工作者项目的发展援助占比逐渐下降,不稳定,且近年来仍在下降。目前仍需开展更多研究,跟踪政府对与社区卫生工作者相关项目的投资,评估投资对方案有效性的影响。

## Резюме

**Цель:**

Оценить уровень и тенденции проектов по оказанию помощи в развитии работников здравоохранения на уровне общин в странах с низким и средним уровнем дохода в период с 2007 по 2017 год.

**Методы:**

Авторы извлекли данные по финансированию проектов поддержки работников здравоохранения (CHW) на уровне общин в 114 странах за 2007–2017 гг. из системы отчетности кредиторов Организации экономического сотрудничества и развития. Были подготовлены сметы для проектов, специально описанных соответствующими ключевыми словами, и для проектов, которые могут включать компоненты по CHW. Авторы проанализировали тенденции оказания помощи в развитии по целям, донорам, регионам и странам-получателям помощи, а также изменение тенденций с течением времени.

**Результаты:**

В период с 2007 по 2017 год общий объем помощи в развитии по проектам CHW составил около 5298,02 млн долл. США, что составляет 2,5% от общего объема помощи в развитии в сфере здравоохранения в размере 209 277,99 млн долл. США. Три крупнейших донора (Глобальный фонд по борьбе со СПИДом, туберкулезом и малярией, Правительство Канады и Правительство Соединенных Штатов Америки) выделили на эти проекты помощи в развитии в общей сложности 4350,08 млн долл. США (82,1%). Страны Африки к югу от Сахары получили в общей сложности 3717,93 млн долл. США, что является самым большим объемом помощи на душу населения за последние 11 лет (0,39 долл. США; общая численность населения: 9426,25 млн). Проекты помощи в развитии, сосредоточенные на лечении инфекционных заболеваний, а также на здоровье матери и ребенка, получили наибольший объем средств за весь период исследования.

**Вывод:**

Доля инвестиций в проекты CHW была небольшой, непостоянной и за последние годы уменьшилась. Необходимы дополнительные исследования по отслеживанию государственных инвестиций в проекты, связанные с CHW, и оценке влияния инвестиций на эффективность выполнения программ.

## Introduction

To attain universal health coverage for the most vulnerable populations and to prepare against future epidemics, such as Ebola, there have been calls for scaling-up community health worker (CHW) programmes.^1^^–^^8^ The aim would be to address the unmet gap of health services caused by geographical, financial and sociocultural barriers in low-resource settings. Country case studies have shown that CHWs can contribute to improving health outcomes for children, pregnant women and other patients.^1^^–^^6^^,^^9^^–^^19^ To be effective, CHWs should be well-trained and well-supervised, given logistical support for the supplies and medications they deliver and provided with appropriate and regular financial incentives.

One of the most important challenges for successfully scaling-up CHW programmes is obtaining sufficient and sustainable funding to support the programme.^3^^–^^5^ A study has estimated that deploying CHWs in rural areas of sub-Saharan African countries requires at least 2.6 billion United States dollars (US$), funded either by national governments or donor partners or both.^20^ Other analyses suggest even higher figures.^5^^,^^21^ Knowing how much has been invested in CHW programmes is important for policy-makers, donors and other stakeholders to make evidence-based planning and budgeting. Our literature review for this study demonstrates that the empirical evidence about existing investments is scant at best.

We aimed to assess the level and trend of development assistance for CHW programmes. International donor contributions play an important role in financing the health sector of low-income countries.^22^ Estimating development assistance for CHW programmes is necessary for establishing a financial baseline for such programmes and identifying funding gaps. Knowing who are the top investors in CHW programmes will help locate potential sources of funds for scaling-up these programmes in the future. Here, we provide a descriptive analysis on the flows of total and per capita development assistance for CHW programmes by donors, recipient regions and recipient countries. We also investigated the priorities of the development assistance for CHWs and estimated per capita aid for CHW disbursed to low- and middle-income countries between 2007 and 2017.

## Methods

### Data sources

We extracted data from the aid activity database from the creditor reporting system of the Organisation for Economic Co-operation and Development’s development assistance committee.^23^ The creditor reporting system database is publicly accessible and has been widely used in aid tracking studies.^24^^–^^30^ The data are reported directly by the governments of the 30 members of the development assistance committee, 65 multilateral organizations (e.g. the World Bank group) or global health initiatives (e.g. the Global Fund to Fight AIDS, Tuberculosis and Malaria), 25 non-development assistance committee countries (e.g. United Arab Emirates) and 28 private donors (e.g. Bill & Melinda Gates Foundation). We downloaded the data used in this study in July 2019. We included 119 countries classified as low- and middle-income by the World Bank in 2015^31^ (available in the data repository).^32^ These were countries that had received aid for CHW projects from 41 donors since 2007 and had available annual data on population and on World Bank region classifications.^33^^,^^34^

### Definitions

We followed the World Health Organization (WHO) definition of CHWs as: health workers who have been trained to some extent, but do not possess a formal professional certificate.^15^ Examples include village health workers, peer supporters, community volunteers and health extension workers. Meanwhile, we were aware of some programmes that aimed for more formalization, accreditation or certification of this role.^35^^,^^36^ We defined development assistance as aid disbursed to projects that support CHW programmes in low- and middle-income countries. In the creditor reporting system data, the term projects refers to programmes and activities that are supported with development assistance.

There were no indicators in the creditor reporting system data on projects for CHWs. Following previous practice,^25^^–^^30^ we constructed a list of keywords (Box 1), based on a review of keywords used in other literature to identify projects for CHW programmes. Our focus was on projects in three health-related sectors provided by the creditor reporting system data: (i) general health; (ii) basic health; and (iii) population policies or programmes and reproductive health.

Box 1 **Keywords used to search for community health worker-related projects in the creditor reporting system**

**Community health worker-targeted projects (lower bound of estimates)**

Accompagnateur, accredited social health activist, ASHA, animator, auxiliary nurse, allied health, barefoot, birth attendant, bridge-to-health team, care group, case coordinator, child health worker, CHW, close-to-community provider, community agent, community aide, community-based practitioner, community case management, community coordinator, community drug distributor, community health care provider, community healthcare provider, community health nurse, community health representative, community health surveyor, community health volunteer, community health care worker, community healthcare worker, community liaison, community nutrition worker, community practitioner, community resource person, community surveillance volunteer, community volunteer, community worker, care group, dame health worker, door-to-door, extension service, extension officer, extension staff, extension worker, family planning agent, family advocate, family health worker, family support worker, family welfare assistant, family welfare worker, female multipurpose health worker, field-based, frontline, grassroots, hard-to-reach, health activist, health aide, health agent, health care agent, health assistant, health auxiliary, health care worker, health coach, health counsellor, health development army, health distributor, health education, health extension, health nurse, health officer, health motivator, health outreach, health promoter/promotor, health surveillance assistant, health visitor, health worker, health volunteer, home-based care, home care, home health, home service, home visit, ICCM, IMCI, intake specialist, integrated community case management, lady health worker, lay aide, lay attendant, lay consultant, lay counsellor, lay health advisor, lay health worker, lay visitor, lay worker, lead mother, LHWs, link worker, malaria agent, malaria and child health worker, maternal and child health worker, medical assistant, midwife, mobile clinic team, mother coordinator, mother leader, navigator, nutrition agent, nutrition counsellor, outreach advocate, outreach case manager, outreach educator, outreach worker, parent liaison, peer advisor, peer counsellor, peer educator, peer health advisor, peer leader, peer supporter, support worker, surveillance volunteer, VHWs, village drug-kit manager, village health, village volunteer, voluntary worker, volunteer.

**Community health worker-inclusive projects (upper bound of estimates)**

All keywords in the community health worker-targeted category above, plus: community-based, community based, community health, community intervention, community participation, community prevention, DOTS.

ASHA: accredited social health activist; CHW: community health worker; DOTS: directly observed treatment, short course; LHW: lady health workers; IMCI: integrated management of childhood illness; ICCM: integrated community case management; VHWs: village health workers. Note: We constructed the list based on a review of keywords used in other literature to identify projects for community health worker programmes.

As some projects on community-based interventions or community health might not have included CHW-related keywords in their project descriptions, we generated two sets of data on development assistance for CHW programmes. To obtain the lower bound of estimates, we calculated aid that targeted CHW projects, as specifically defined by the keywords in community health worker-targeted projects listed in Box 1. To obtain the upper bound of estimates, we calculated aid that included both CHW-targeted projects and community intervention projects that were not defined by these keywords, but which included keywords indicating CHW components, as described in the Community health worker-inclusive projects listed in Box 1.

Some projects may have included other components that were not related to CHWs. In this case, we were not able to allocate funds to the CHWs component due to lack of information in the data. We therefore considered aid to those projects as investments in CHW projects. Details of the keyword search and our validation strategy are available in the data repository.^32^

### Analysis

We used actual disbursements to estimate development assistance for CHWs between 2007 and 2017. This time frame allowed us to avoid the issue of missing disbursement data in the creditor reporting system: the missing rate of total disbursements in three health sectors was approximately 23% on average between 2000–2006.^28^ The completeness of disbursement data has remained at almost 100% since 2007.^37^ A total of 81.0% (US$ 196 539.00 million) of US$ 242 598.30 million development assistance for health during this period was in the form of grants and 5.7% (US$ 13 897.26 million) as concessional loans. We report all disbursements in 2017 US$.

We estimated levels and trends of development assistance for CHWs over 2007–2017 in both total and per capita spending at the global, regional and country levels. We identified the top 10 donors of development assistance for the CHW projects and the 10 CHW-targeted projects that had received the largest disbursements. When we calculated country-level estimates, we excluded projects that were classified as going to a region only since no further information was provided on the amounts provided to each country in that region.

In the three health sectors that we included in our analysis (general health; basic health; and population policies or programmes and reproductive health), projects were categorized by the creditor reporting system into 17 primary purposes, such as sexually transmitted diseases including human immunodeficiency virus and acquired immune deficiency syndrome (HIV/AIDS), basic nutrition, and so on.^37^ Further details are available in the data repository.^32^ We excluded 98 projects whose primary purpose was research, such as randomized trials. After excluding one purpose (medical research), we finally assessed the allocation of development assistance for CHW-targeted projects across the 16 purposes, with 4600 CHW-targeted projects and 10 363 CHW-inclusive projects. We analysed development assistance for projects that aimed to improve the health-related millennium development goals (MDGs)^38^ and grouped them into two areas: (i) HIV, tuberculosis and malaria; and (ii) child and maternal health, including projects on family planning, reproductive health, basic nutrition, and child, maternal and newborn health. We used variables on the primary purposes of a project and adopted keywords from previous studies to further identify the projects in these two areas (available in the data repository).^32^ Note that development assistance for CHW projects in these two areas are not mutually exclusive; a project targeting infectious diseases may also include support for child and maternal health.

In this article we present the development assistance for CHW-targeted projects in 114 countries. The results of development assistance for CHW-inclusive projects in 119 countries are available in the data repository.^32^

## Results

**Fig. 1:**
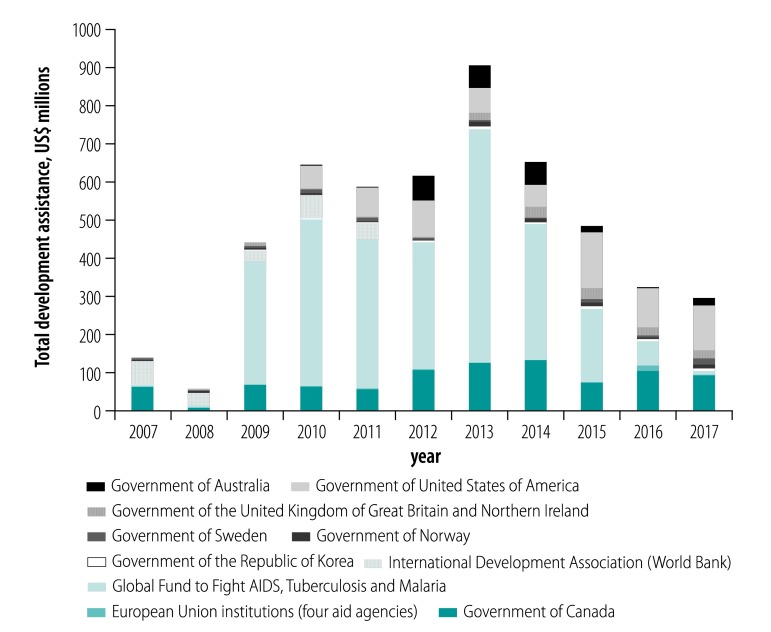
Top 10 funders of aid to community health worker-targeted projects, 2007–2017

Between 2007 and 2017, an estimated total of US$ 5298.02 million was disbursed to 4600 CHW-targeted projects. The amount funded annually had an upward trend from 2008 (US$ 68.88 million) to 2013 (US$ 916.56 million), and then decreased from 2014 (US$ 665.16 million) to 2017 (US$ 307.27 million; Table 1). The largest increase occurred between 2008 and 2009, from US$ 68.88 million to US$ 451.20 million, largely due to increased funds from the Global Fund (from US$ 5.91 million in 2008 to US$ 322.81 million in 2009) and from the government of Canada (from US$ 8.65 million in 2008 to US$ 68.72 million in 2009). The decline after 2013 was mainly driven by reduction of investments by the Global Fund, from US$ 609.82 million in 2013 to US$ 7.62 million in 2017 (Fig. 1).

**Table 1:** Aid disbursements to community health worker-targeted projects in 114 low- and middle-income countries, 2007–2017

Year	Aid disbursements, US$	
	Total allocated to health	Allocated to community health worker-targeted projects (% of total)
2007	13 449 724 134	152 968 411 (1.14)
2008	14 829 155 928	68 878 278 (0.46)
2009	16 958 334 753	451 198 160 (2.66)
2010	18 199 857 966	670 183 967 (3.68)
2011	18 619 062 848	598 481 199 (3.21)
2012	19 253 920 673	628 446 202 (3.26)
2013	21 290 831 362	916 556 626 (4.30)
2014	20 083 970 589	665 164 862 (3.31)
2015	20 809 549 322	500 892 226 (2.41)
2016	21 579 902 568	337 989 701 (1.57)
2017	24 203 678 042	307 265 350 (1.27)
**Total**	**209 277 988 185**	**5 298 024 982 (2.53)**

US$: United States dollars.

Notes: We extracted data from the aid activity database from the creditor reporting system of the Organisation for Economic Co-operation and Development’s development assistance committee.^23^

US$: United States dollars.

Notes: We extracted data from the aid activity database from the creditor reporting system of the Organisation for Economic Co-operation and Development’s development assistance committee.^23^ US$ in year 2017.

Of the total US$ 209 277.99 million development assistance disbursed to health over the period of the study, the percentage invested in CHW-targeted projects averaged 2.5% (US$ 5 298.02 million). The percentage ranged from 0.5% in 2008 (US$ 68.88 million for CHW projects out of 14 829.16 million to health) to 4.3% in 2013 (US$ 916.56 million out of 21 290.83 million total; Table 1).

### Estimates by donor

Among the 37 donors that disbursed funds to CHW-targeted projects, the top 10 and their annual contribution are presented in Fig. 1. The top three donors during the study period were the Global Fund (US$ 2 718.09 million), government of Canada (US$ 900.39 million), and government of the United States of America (US$ 731.60 million). Altogether they provided 82.1% of total development assistance for CHW-targeted projects.

### Estimates by region

**Fig. 2:**
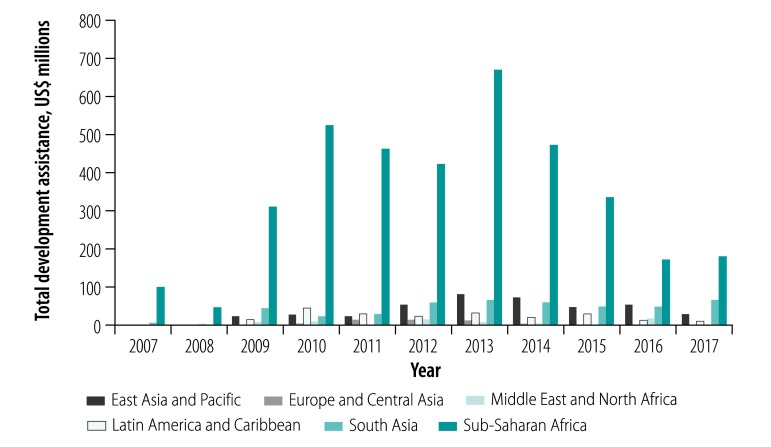
Aid disbursements to community health worker-targeted projects by World Bank region, 2007–2017

Among the six regions analysed (World Bank classifications), sub-Saharan African countries consistently received the largest amount of development assistance (US$ 3 717.93 million in total over the 11 years), accounting for 70.2% of total development assistance disbursed to CHW-targeted projects (Fig. 2). When taking population size into consideration (estimated total population over 11 years: 9 426.25 million), sub-Saharan African countries had the largest per capita development assistance (US$ 0.39) during the study period.

US$: United States dollars.

Notes: We extracted data from the aid activity database from the creditor reporting system of the Organisation for Economic Co-operation and Development’s development assistance committee.^23^ We used World Bank region classifications.^31^ US$ in year 2017.

### Estimates by purpose

Among the 16 health purposes labelled by the creditor reporting system data, the projects with a focus on three infectious diseases (HIV, tuberculosis and malaria) and child and maternal health received the most funds. For example, the category sexually-transmitted disease control including HIV/AIDS received the largest amount (US$ 2 061.14 million) and percentage of total disbursements (38.9%) during the study period, followed by malaria control (US$ 1 047.46 million; 19.8 %) and reproductive health care (US$ 490.85 million; 9.3%; Table 2).

**Table 2:** Aid disbursements to community health worker-targeted projects in 114 low- and middle-income countries by health purpose, 2007–2017 (in 2017 US$)

Health purpose	No. of projects	Aid disbursements, US$ (% of total)
Sexually transmitted disease control including HIV and AIDS	672	2 061 141 216 (38.9)
Malaria control	224	1 047 456 209 (19.8)
Reproductive health care	629	490 852 296 (9.3)
Basic health care	701	411 216 330 (7.8)
Tuberculosis control	150	226 866 180 (4.3)
Health personnel development	397	211 871 292 (4.0)
Health policy and administrative management	414	188 310 443 (3.6)
Basic nutrition	222	166 084 976 (3.1)
Personnel development for population and reproductive health	165	109 434 420 (2.1)
Family planning	133	100 643 630 (1.9)
Infectious disease control	99	93 732 715 (1.8)
Health education	318	61 616 914 (1.2)
Basic health infrastructure	189	51 214 423 (1.0)
Medical education and training	135	39 184 445 (0.7)
Population policy and administrative management	41	20 971 755 (0.4)
Medical services	111	17 427 738 (0.3)
**Total**	**4 600**	**5 298 024 982 (100.0)**

AIDS: acquired immune deficiency virus; HIV: human immunodeficiency virus; US$: United States dollars.

Note: We extracted data from the aid activity database from the creditor reporting system of the Organisation for Economic Co-operation and Development’s development assistance committee.^23^

The 10 projects that received the largest disbursements during the period were in sub-Saharan African countries. Eight of them were funded by the Global Fund. The other two donors were the International Development Association of the World Bank and the government of the United States. Seven projects had a focus on HIV, two on malaria and one on child and maternal health (Table 3).

**Table 3:** Top 10 community health worker-targeted projects receiving the largest aid disbursements, 2007–2017 (in 2017 US$)

Project title	Recipient country	Disbursements, US$	Donor
Sustaining the momentum: the march towards universal access to HIV and AIDS services in United Republic of Tanzania	United Republic of Tanzania	411 429 632	Global Fund
HIV/AIDS prevention and control	Ethiopia	383 825 888	Global Fund
Scaling-up and sustaining anti-malaria interventions in Ethiopia	Ethiopia	269 869 248	Global Fund
National response to HIV/AIDS in Malawi	Malawi	131 990 000	Global Fund
Second health systems development (child and maternal health)	Nigeria	127 552 656	International Development Association
Scaling up the fight against HIV/AIDS in Namibia	Namibia	104 299 232	Global Fund
Accelerating access to prevention, treatment, and home-based care for malaria and increasing the access to affordable ACTs in the private sector	Ghana	96 355 600	Global Fund
HIV/AIDS treatment project – HIV/AIDS	South Africa	94 448 896	Government of the United States
Strengthening and expanding the Western Cape HIV/AIDS prevention, treatment and care programmes	South Africa	77 013 792	Global Fund
Seventh call for proposals HIV and AIDS	Eswatini	75 822 072	Global Fund

ACTs: artemisinin-based combination therapies; AIDS: acquired immune deficiency virus; Global Fund: Global Fund to Fight AIDS, Tuberculosis and Malaria; HIV: human immunodeficiency virus; US$: United States dollars.

Note: We extracted data from the aid activity database from the creditor reporting system of the Organisation for Economic Co-operation and Development’s development assistance committee.^23^

### Estimates by country

Among the 25 countries that received more than US$ 0.50 per capita aid for CHW over the 11 years, 12 were low-income countries and three were upper-middle income countries (Fiji, Guyana and Namibia). The five countries that received the largest per capita development assistance over the study period were Namibia: US$ 7.85 (US$ 139.19 million in 11 years to population of 17.73 million in 11 years), Eswatini: US$ 6.90 (US$ 82.00 million in 11 years to population of 11.89 million in 11 years), Fiji: US$ 5.42 (US$ 28.28 million in 11 years to population of 5.22 million in 11 years), Guyana: US$ 3.70 (US$ 25.24 million in 11 years to population of 6.81 million in 11 years) and Gambia: US$ 2.27 (US$ 34.08 million in 11 years to population of 15.00 million in 11 years; available in the data repository^32^.

## Discussion

Our analysis of data from the creditor reporting system on aid disbursed to projects that supported CHWs between 2007 and 2017 revealed two main findings. First, development assistance for these projects accounted for less than 3% of total development assistance for health during the period, with a decrease since 2013 mainly due to the reduction of Global Fund investments in these projects. Second, the focus of CHW-targeted projects was mostly on achieving the health-related MDGs (HIV, tuberculosis, malaria and child and maternal health) and most of the assistance was disbursed to sub-Saharan African countries. This finding suggests that donors’ investment in CHWs was in line with efforts towards achieving the health-related MDGs in the region.

There is increasing evidence of the effectiveness of CHWs in contributing to improved health outcomes for children and women. For example, a review of efficacy studies indicated that CHWs could reduce maternal mortality by 42–78%.^39^ A review of studies on the efficacy of CHWs’ prescribing antibiotics for treatment of pneumonia (one of the leading causes of mortality among infant and children younger than 5 years) indicated that under-5 mortality could be reduced by 13–60%.^40^ The long-term impacts of CHWs on social and economic development, including empowering women and increasing jobs especially in remote poor and rural areas, could result in a positive economic return, as high as 10:1.^3^ There is now a broad global consensus that CHWs are essential for efforts to achieve the 2030 health-related sustainable development goal (SDG) targets, including the achievement of universal health coverage.^7^^,^^8^^,^^41^^–^^44^

Our study shows that the average annual amount of development assistance for CHW-targeted projects in sub-Saharan African countries over 2007–2017 was US$ 0.34 billion. It has been estimated that the governments of sub-Saharan African countries spent US$ 0.4 billion of their own funds on CHWs.^21^ The sum of these two estimates falls far short of the estimates required for CHWs to be minimally effective (US$ 2.6–3.1 billion^5^^,^^20^^,^^21^), highlighting an urgent need to build national political and financial support for CHWs in the region.

In the short term, however, low-income countries are not able to fully support a national CHW programme with domestic public spending alone. To illustrate the importance of donor investment in low-income countries we made a case study of Rwanda. Box 2 shows that the country’s CHW programmes, which played an essential role in provide basic care for child and maternal health, were mainly funded by foreign aid. The programme made important contribution to reaching the country’s health MDGs,^3^ even with less than half the recommended level of per capita investment (US$ 3.25 versus US$ 6.86).^20^ In our study, among the 25 countries that received the largest per capita aid for CHWs over the 11 years, only 12 were low-income countries.

Box 2 **Case study: financing community health worker programmes in Rwanda**

Established in 1995, Rwanda’s CHWs have become an essential part of the Rwandan health system and have played an important role in expanding service coverage in Rwanda, especially in remote rural areas.^3^ According to a government report in 2016, about 45 000 CHWs served 11 million people.^45^ The CHWs were providing services reliably in each village, including health promotion (e.g. healthy living), disease prevention (e.g. bed-net utilization) and treatment (e.g. treatment of malaria). Major activities included promoting child and maternal care (e.g. antenatal home visits and treating or referring young children with diarrhoea or malnutrition) and treating tuberculosis and malaria.

Health resource tracking data in Rwanda provides project-level expenditure information by funding sources.^46^ We used data that were available in fiscal years 2010–2011 and 2011–2012 to estimate the spending on CHWs by the Rwanda government and foreign donors in the two years. Funds were calculated in 2010 US$.

**Funding for CHWs in fiscal year 2010–2011^a^**

• From bilateral or multilateral donors: US$ 14 695 657 (83.1%)

• From international NGOs: US$ 2 714 026 (15.3%)

• From government of Rwanda: US$ 273 182 (1.5%)

• Total: US$ 17 682 865 (100.0%)

• Per capita (population: 10 516 000: US$ 1.68

**Funding for CHWs in fiscal year 2011–2012^a^**

• From bilateral or multilateral donors: US$ 33 299 698 (95.0%)

• From international NGOs: US$ 1 504 775 (4.3%)

• From government of Rwanda: US$ 240 777 (0.7%)

• Total: US$ 35 045 250 (100.0%)

• Per capita (population: 10 789 000): US$ 3.25

Foreign donors (bilateral, multilateral and international nongovernment organizations) were the major funding contributors to CHWs. In fiscal year 2011–2012, for example, 95.0% of funding for the CHWs (US$ 33 299 698 out of 35 045 250) were from bilateral or multilateral donors.

CHWs: community health workers; NGOs: nongovernmental organizations; US$: United States dollars.^a^ We used the population in calendar years 2011 and 2012 in calculations, which could lead to an underestimate of per capita costs, as the population of Rwanda was increasing between 2010 and 2012. Population data were obtained from the World Health Organization.^47^

A marked fluctuation of the development assistance for CHW-targeted projects over time was mainly driven by changes in investments from one or two donors. Relying on a few donors poses challenges for maintaining stable funding levels and effective long-term budgeting for recipient countries.

Our study had limitations. First, the creditor reporting system data do not include aid from many private nongovernmental organizations as well as from emerging economies such as China. To address this issue, we will conduct future searches on the websites of nongovernmental organizations that are known for supporting CHWs in recent years (e.g. the Clinton Health Access Initiative and Global Health Workforce Alliance). Second, our identification strategy relied on keywords, which were not able to capture all projects on development assistance for CHW programmes. However, we estimate that approximately 5% of projects were missed, indicating that our findings are robust (available in the data repository).^32^ Third, the accuracy of our data could suffer from errors or incompleteness in the reporting of the characteristics of the projects supported through development assistance for health. Fourth, for projects with multiple foci, we were unable to determine how much money went to each focus, so we considered that the total funds went to support CHW programmes. We may therefore have overestimated the amounts donated for CHW programmes. Fifth, when estimating development assistance for CHWs at the country level, project funds that were disbursed to an entire region were not included because of a lack of information about how to allocate funds to each country.

Several lessons can be drawn from our study. First, our finding that less than 3% of total development assistance for health was targeted on CHW programmes indicates that there is an opportunity to improve the effectiveness of this assistance by investing more in programme expansion and strengthening. This finding is important as there is extensive evidence about the effectiveness of CHWs in improving maternal, neonatal and child health, including control of malaria and expanding contraceptive access.^48^^–^^51^ There is a continuing need to reduce the number of readily preventable deaths in these population groups to achieve the global goal of eliminating preventable maternal and child mortality by 2030.^52^^,^^53^

Second, it is concerning that development assistance for CHWs has been decreasing since 2014 and only accounted for 1.27% of total development assistance for health in 2017. The reduction was due to a decrease in support from the three main donors of the development assistance for CHWs. There needs to be more stability of CHW programme assistance over time, support from a broader set of donors and a broader focus of the support beyond high-priority infectious diseases in the era of SDGs. 

Third, further research is needed into the amount of investment going into CHW programmes – both from external development assistance as well as from governments’ own internally generated funds – and the proportion of investment going for CHW programmes relative to other areas of investment in health. Investments in CHW projects is an important but neglected area of research.

Finally, we need evidence of the effectiveness of development assistance for CHWs in terms of both the role of aid for these programmes in improving population health outcomes and the best ways to support the programmes. The future research would include monitoring investments in CHW programmes and assessing their impact on CHW programme effectiveness. This information will help policy-makers and other stakeholders plan for long-term financing strategies for scaling-up and sustaining CHW programmes.
